# Quantitative analysis of prion disease using an AI-powered digital pathology framework

**DOI:** 10.1038/s41598-023-44782-4

**Published:** 2023-10-18

**Authors:** Massimo Salvi, Filippo Molinari, Mario Ciccarelli, Roberto Testi, Stefano Taraglio, Daniele Imperiale

**Affiliations:** 1 Biolab, PoliTo(BIO)Med Lab, Department of Electronics and Telecommunications, Politecnico di Torino, Corso Duca degli Abruzzi 24, 10129 Turin, Italy; 2 SC Medicina Legale, ASL Città di Torino, Turin, Italy; 3 SC Anatomia Patologica, ASL Città di Torino, Turin, Italy; 4 SC Neurologia Ospedale Maria Vittoria & Centro Diagnosi Osservazione Malattie Prioniche, ASL Città di Torino, Turin, Italy

**Keywords:** Biomedical engineering, Neurological disorders, Engineering

## Abstract

Prion disease is a fatal neurodegenerative disorder characterized by accumulation of an abnormal prion protein (PrPSc) in the central nervous system. To identify PrPSc aggregates for diagnostic purposes, pathologists use immunohistochemical staining of prion protein antibodies on tissue samples. With digital pathology, artificial intelligence can now analyze stained slides. In this study, we developed an automated pipeline for the identification of PrPSc aggregates in tissue samples from the cerebellar and occipital cortex. To the best of our knowledge, this is the first framework to evaluate PrPSc deposition in digital images. We used two strategies: a deep learning segmentation approach using a vision transformer, and a machine learning classification approach with traditional classifiers. Our method was developed and tested on 64 whole slide images from 41 patients definitively diagnosed with prion disease. The results of our study demonstrated that our proposed framework can accurately classify WSIs from a blind test set. Moreover, it can quantify PrPSc distribution and localization throughout the brain. This could potentially be extended to evaluate protein expression in other neurodegenerative diseases like Alzheimer's and Parkinson's. Overall, our pipeline highlights the potential of AI-assisted pathology to provide valuable insights, leading to improved diagnostic accuracy and efficiency.

## Introduction

Prion diseases, also referred to as transmissible spongiform encephalopathies (TSEs), are an incurable group of transmissible neurodegenerative conditions characterized by the presence of aggregates of the pathological isoform of the cellular prion protein (PrPSc)^1,2^. Once PrPSc is generated, a seeded-conversion mechanism begins, and the aberrant protein acts as a template for the recruitment and conversion of healthy proteins with the same amino acid sequence^1,3^.

**Figure 1:**
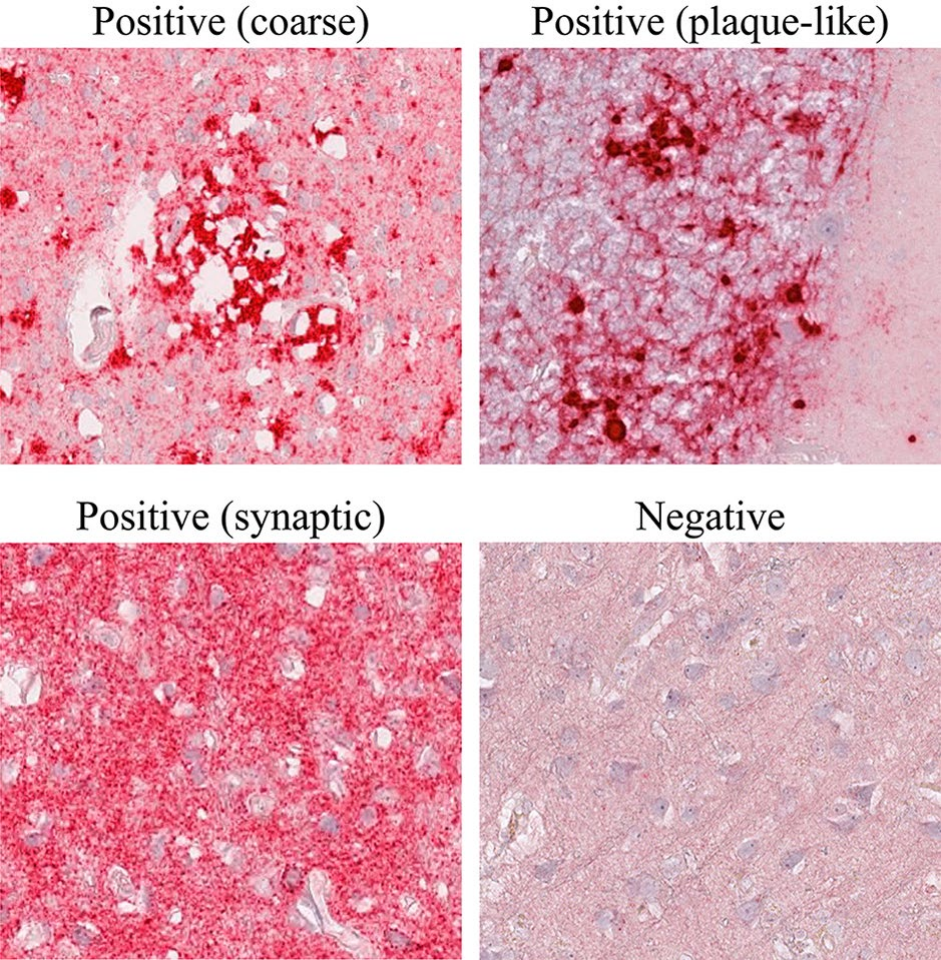
Histopathological patterns used to evaluate prion disease. Positive patterns demonstrate an accumulation of the abnormal prion protein, resulting in a more granular texture of the image. In contrast, negative patterns show a normal distribution of protein.

In 2018, the Centers for Disease Control and Prevention (CDC) established that the standard pathological techniques for diagnosing prion diseases include immunocytochemistry, Western Blot, and histopathological analysis to detect scrapie-associated fibrils^4^. The diagnosis is usually made post-mortem by performing an autopsy, which allows for easy examination of the nervous tissue from various regions of the central nervous system (CNS) and accurate detection of features such as astrocytosis, neural loss, spongiosis, and PrPSc presence as evidenced by immunohistochemistry^1^. Histopathological patterns require immunolabeling for visualization and can be classified into three categories^5,6^ as shown in Fig. 1. Although none of these patterns identifies a specific phenotype, their presence and location are valuable for disease subtype classification. However, the identification and characterization of certain prion protein aggregates, particularly synaptic deposits, pose challenges in immunohistochemical staining.

The diagnostic process for prion diseases is time-consuming, and physicians can be overwhelmed by the workload. Therefore, an automated tool that recognizes WSIs with PrPSc aggregates would speed up the procedure and enable pathologists to work more efficiently. Furthermore, the assessment of prion disease involves examining various regions within the brain, thereby increasing the time required for pathologists. Moreover, pathologists can only analyze one section at a time, posing a challenge in reconstructing the distribution of the prion protein throughout the entire brain. Lastly, in situations where comprehensive sampling is necessary, a tool that quantifies protein expression and localization across the entire brain would be highly beneficial for future research purposes.

Over the past two decades, pathology has undergone a technological revolution similar to what radiology experienced in the previous century. The catalyst for this process was the widespread adoption of whole slide images (WSIs), which are digital reproductions of slides generated by scanners. Unlike traditional microscopy, WSIs offer several advantages due to their digital nature^7–10^. Sharing WSIs is easier and faster than sharing physical slides, resulting in more efficient and accurate diagnoses by multiple pathologists^8,10^. In addition, the increased availability of WSIs has made them valuable in training future experts^8^. Moreover, WSIs are well-suited for quantitative analysis using artificial intelligence (AI) algorithms. As a result, the use of machine learning and deep learning in tasks such as segmentation, classification, and prognosis has exponentially increased in recent years, leading to numerous research papers^7–10^.

In this context, it is important to emphasize the significance of standardization and quality control in the field of digital pathology. Optimal slide preparation, accurate staining techniques, and high-quality digitalization processes are essential factors that contribute to the development of reliable and robust AI tools. Standardization ensures consistency in slide preparation, minimizing variations that can affect the accuracy and reproducibility of AI algorithms. Accurate staining techniques are crucial for highlighting specific features and biomarkers in the tissue samples, enabling AI algorithms to effectively identify and analyze them. Additionally, high-quality digitalization processes ensure that the digitized slides capture the essential details and characteristics needed for accurate analysis by AI algorithms.

AI-based systems are increasingly popular due to their ability to learn from data and perform tasks at a human cognitive level^11–13^. In the medical field, these algorithms can offer significant support to physicians by automatically performing time-consuming tasks, speeding up medical analysis^14–16^. They can also extract information beyond what the human eye can see and provide it to the physician, reducing the error rate^7^. AI technology has shown remarkable results in tasks such as lesion identification and labeling^17^, target area segmentation, 3D image reconstruction^18^, image classification^19^, and processing. Furthermore, an AI system can quantitatively examine images, while expert microscopic analysis is qualitative and prone to intra- or inter-operator variability. Therefore, a second opinion based on numerical data could support the pathologist and significantly reduce misclassifications^20^.

The assessment of prion disease using AI poses various challenges that must be addressed, such as obtaining a wide and high-quality dataset and manual segmentations, managing the non-Boolean nature of the diagnosis^21^, interpreting the results, and handling the large dimension of WSIs^22^. To create a usable system, WSIs must accurately represent the casuistry, as the presence of artifacts or suboptimal coloring could lead to diagnostic errors and hinder the development of a robust system. Manual segmentations are essential for teaching the system to differentiate between different labels but collecting them is time-consuming and subject to problems related to qualitative evaluation, such as inter- and intra-operator variability and uncertainty. Nevertheless, manual segmentations are crucial for obtaining accurate results because the algorithm considers the expert's knowledge as the gold standard. Another challenge is related to the interpretation of the results: although the diagnosis of prion disease may be binary, descriptive terminology that accounts for the clinical context, pathologist's perception, and experience is used when exploring the disease phenotype. Replicating this process is not trivial, especially since there is no clear way to explain why the algorithm has made a certain decision, making the results difficult to interpret^23^. Finally, digital slides typically contain hundreds of millions or even billions of pixels, while neural networks take relatively small images as input. This necessitates a patch extraction phase, which involves extracting smaller regions from the original WSI. However, this process could result in the loss of relevant information that could be obtained from an overall view of the image^7^.

In the last decade, AI has been widely used for brain image classification of Alzheimer’s Disease (AD) and Parkinson’s Disease (PD), which represent the most studied neurodegenerative diseases^24–26^. In digital pathology, Convolutional Neural Networks (CNNs) have been proposed for classifying slides from patients with glioma^27^ or tauopathy^28^. Recently, researchers have also used AI to study prion diseases: Bhakta et al.^29^ investigated potential correlations between behavioral and environmental factors and the disease, while and Bizzi et al.^30^ proposed AI-based diagnostic methods for prion diseases from magnetic resonance imaging. However, both methods required manual effort to extract input features. Currently, detecting PrPSc accumulation through post-mortem tissue analysis is necessary for definitive prion disease diagnosis, and no research has been published on using AI to classify WSIs for prion disease.

This work aims to develop an AI-based framework that employs both machine learning (ML) and recent deep learning (DL) methods for accurate classification of WSIs as positive or negative for prion disease, and to quantify the distribution of prion proteins across entire tissue sections. The main contributions of this paper can be summarized as follows:

- We propose the first AI-based framework for the evaluation of prion diseases in digital pathology images. Our algorithm employs ML-based and DL methods to accurately classify prion diseases using immunohistochemistry (IHC) slides. A comprehensive comparison is also carried out between these two techniques to find the best method in terms of accuracy and computational efficiency.
- We present a new segmentation network that combines a multi-scale features extraction technique (UPerNet) with a local adaptive attention mechanism (TWINS) to accurately segment PrPSc aggregates. The proposed segmentation network outperforms existing ML methods in the identification of histological patterns associated to prion disease.
- We develop a smart approach for patch extraction during inference that dramatically reduces computational times. Our approach is specifically designed to extract patches only from areas of tissue that are likely to contain PrPSc aggregates. By avoiding the extraction of unnecessary patches, our approach significantly increases the efficiency and speed of the overall analysis, with improvements of up to 700%.
- Our method enables the quantification of PrPSc distribution across entire tissue sections, allowing for the reconstruction and quantification of protein distribution throughout the entire brain. Such an approach can be easily extended to the quantification of other immunohistochemical proteins in different neurodegenerative diseases.

This paper is structured as follows: section "Materials and methods" provides a comprehensive overview of the proposed method, while section "Results" details the experimental results. Finally, sections "Discussion" and "Conclusion" offer a thorough discussion of the overall work.

## Materials and methods

### Dataset

The dataset consisted of 41 patients, with two WSIs provided for 23 of them, referring to the cerebellum and occipital lobe of the cerebral cortex. For the remaining patients, 13 had only cerebellum WSIs and 5 had only occipital cortex WSIs. Overall, the dataset comprised 64 digital slides, with 36 for the cerebellum and 28 for the occipital lobe. The data utilized for this study originates from the "Piedmont Reference Center for Diagnosis and Monitoring of Prion Diseases (DOMP)." The DOMP Center, operating since 2002, is dedicated to institutional diagnostic and monitoring purposes related to prion diseases. The slides were retrieved from archived paraffin-embedded tissue blocks of prion disease cases deceased in 2002–2008 period.

Two different scanners were used to digitize the slides in this study. The Ventana DP200 scanner, manufactured by Roche, was used to scan 30 WSIs, while the remaining slides were scanned using the Aperio T2 scanner (Leica System). By using two different scanners, we aimed to introduce additional variability into the dataset, mimicking the variability that would be expected in a real-world clinical setting. This increased heterogeneity is beneficial because it better reflects the diversity of images that may be encountered in clinical practice, improving the generalizability of our findings, and ensuring that any algorithms or models developed on this dataset will be better suited to real-world scenarios.

Tissue PrPSc deposits were identified by immunohistochemistry with the 12F10 antibody^31^ after pretreatment with 30-min hydrate autoclaving at 121 °C and 5 min 96% formic acid to eliminate the cellular form of prion protein. The immunolabeling of sections was achieved using the avidin–biotin-complex (ABC) method and the Fast Red chromogen in an automated immunostaining machine (Leica Bond, Leica Biosystems Nussloch GmbH, Germany). This allows protein aggregates to be highlighted in positive subjects, resulting in red staining of the pathological tissue. Out of the 64 WSIs in the dataset, a total of 48 were labeled as positive for prion disease by an expert pathologist (D.I.), while the remaining 16 were negative controls. The positive WSIs displayed clear and distinct histopathological patterns associated with prion diseases, such as protein aggregates and neuronal loss, while the negative WSIs did not exhibit any such patterns. The same experienced pathologist manually annotated the regions of WSI that exhibited PrPSc deposits compared to the negative areas.

The WSIs were divided into three distinct subsets: the training and validation sets, which were used to construct and train the models, and a blind test set which served to validate the entire system. The numerical breakdown of each subset in terms of patients, WSIs, and tiles is presented in Table 1, providing an overview of the dataset and the distribution of data used for the development and validation of the system.

**Table 1:** Dataset composition.

Subset	# Patients	# WSI	# Patches
Train set	33	49	16,833
Validation set	5	9	3302
Test set	3	6	3636

To prevent bias and ensure balance, the dataset was divided into subsets for training, validation, and testing. The training set included 33 patients and 49 slides, while the validation and test sets included 5 and 3 subjects, comprising 9 and 6 slides, respectively. Partitioning was carried out based on anatomical regions, histopathological phenotypes, and labels. Due to the large size of the WSIs, the expert operator manually labelled positive and negative patterns to train the AI system. A sliding window was used to extract regions of size 512 × 512x3 at 20 × magnification, which allowed for the observation of patterns at a spatial scale perceptible to the human eye. To prevent dataset imbalance, no more than 500 patches were extracted per slide. More details about patch extraction are reported in the Supplementary Material.

**Figure 2:**
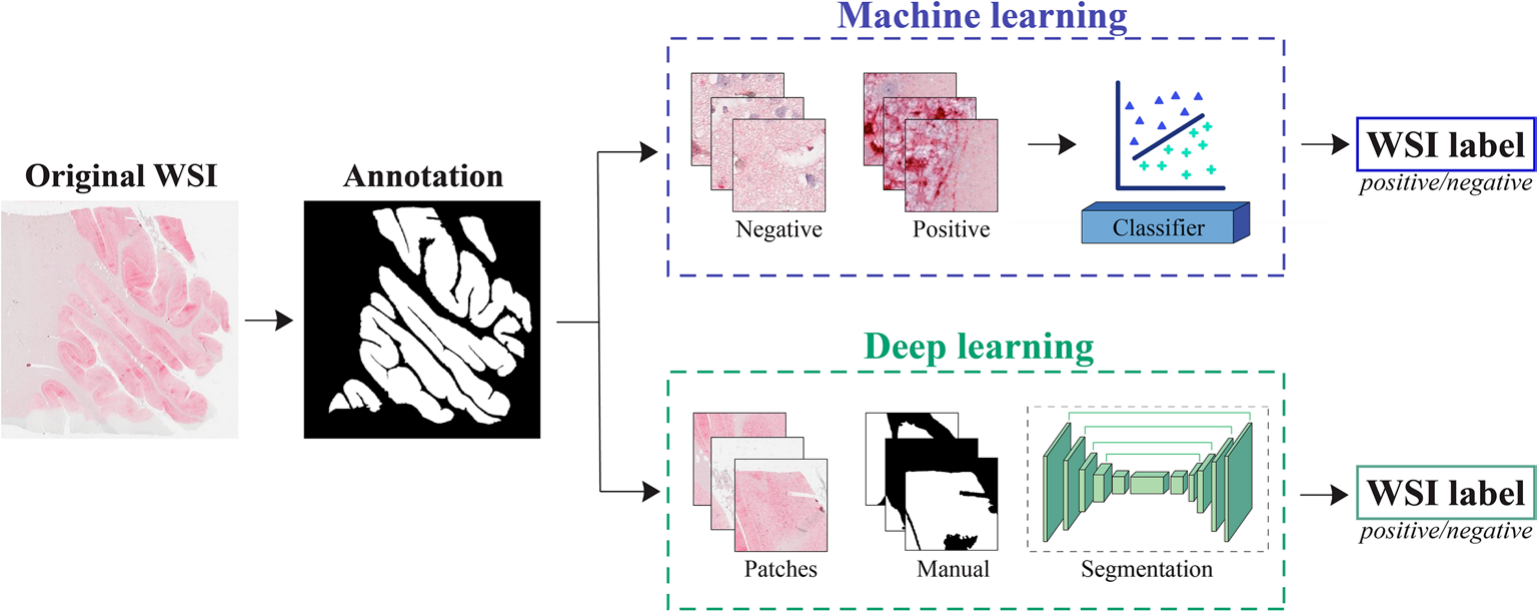
Proposed pipeline for detecting prion diseases in whole-slide images using two different approaches. The first approach relies on machine learning, which classifies each patch in the WSI, while the second approach employs deep learning for semantic segmentation. In both approaches, a patch aggregation strategy is employed to determine whether the entire WSI is positive or negative for prion diseases.

The aim of this study is to distinguish between positive and negative histopathological patterns of prion disease using two different methods: machine learning and deep learning. The machine learning approach involves extracting handcrafted features and performing a classification task to identify PrPSc aggregates in the input patch. On the other hand, the deep neural network can automatically extract features and perform pattern segmentation to determine whether individual pixels belong to the patterns. The key difference between the two approaches lies in their feature extraction and learning paradigm (Fig. 2).

### Feature extraction and classification using machine learning

**Figure 3:**
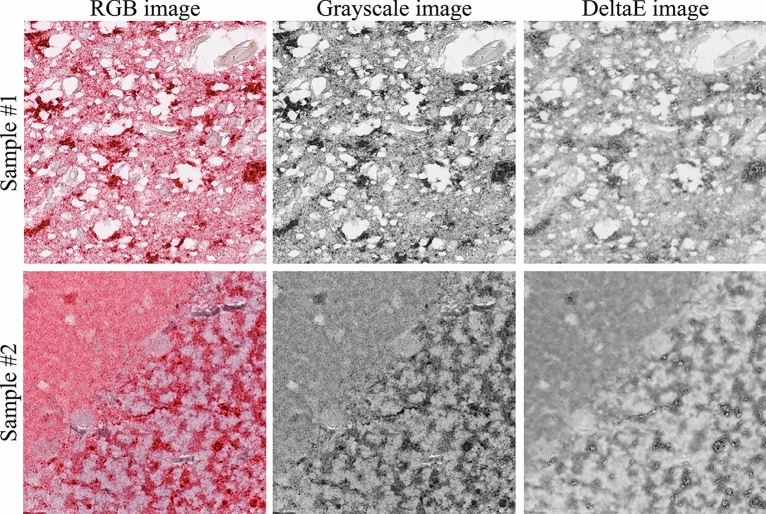
Comparison between the traditional grayscale image and the proposed DeltaE image. DeltaE image effectively reduces staining variability while maintaining the patch's structural content.

To perform feature extraction, we take advantage of texture analysis (TA), a subfield of radiomics that converts images into analyzable data to support decision-making^32,33^. Since TA works on single-channel images, we converted patches from RGB to grayscale. However, this conversion led to a loss of information, as different colors with similar brightness were converted to very similar gray tones (Fig. 3). To mitigate this issue, we explored an alternative single-channel image approach called the DeltaE transform. As illustrated in Fig. 3, the DeltaE transform successfully reduced staining variability while retaining the informative content of the patch.

The DeltaE transform involves converting the RGB image to the LAB color space. Within this color space, there is a linear relationship between various colors, allowing for precise measurement of the distance between any two tones through the calculation of the Euclidean distance. In this application, we used the primary red (RGB triplet: [255, 0, 0]) as our reference color.

1 $$ DeltaE = \sqrt {\left( {L_{i} - L_{r} } \right)^{2} + \left( {a_{i} - a_{r} } \right)^{2} + \left( {b_{i} - b_{r} } \right)^{2} } $$

In Eq. 1, the index *i* denotes the values of the i-th pixel, whereas *L*_*r*_, *a*_*r*_, and *b*_*r*_ correspond to the LAB values of the reference color. Pixels that are closer to the reference color will appear darker due to the smaller distance, while those farther away will appear lighter due to the larger distance in Euclidean space. Since DeltaE values may exceed 255, they are first normalized between 0 and 1 before being converted to integer format. The minimum and maximum values of the DeltaE transforms in the training set were used to determine the lower and upper limits, respectively.

Texture analysis employs mathematical techniques to analyze the intensity and position of pixels within an image. Texture refers to the repetition of patterns and elements within a specific region, which possess consistent perceptual characteristics while the term ‘position’ refers to a group of adjacent pixels with tonal or local properties. A total of 169 texture features were extracted using first- and second-order statistical methods^34,35^ from both the traditional grayscale image and the DeltaE transform.

First-order statistics evaluate the overall distribution of pixel intensities in the image^34^, while second-order statistics consider the spatial interactions of gray levels. The most widely used matrices for second-order statistics are the gray-level co-occurrence matrix (GLCM), gray-level run length matrix (GLRLM)^36^, and local binary pattern (LBP)^37^. GLCM quantifies how many times two gray levels are within a certain distance of each other, and GLRLM evaluates the distribution of gray levels on a larger scale. LBP explores the neighborhood of a pixel and assigns a value to the pixel under consideration in the LBP matrix. The parameters extracted from these matrices are used to calculate various features such as: contrast, dissimilarity, homogeneity, local energy, correlation, second angular momentum, local entropy, short-run emphasis, long-run emphasis, gray level nonuniformity, run length nonuniformity, run percentage, energy, and entropy.

The extracted features were normalized using either min–max scaling normalization or z-score normalization. To reduce the number of dimensions in the feature space, principal component analysis (PCA)^38^ was used. PCA identifies the most informative features that contribute the most to the variance in the data. Additionally, a parallelized variant of the minimum-Redundancy Maximum-Relevance (mRMR) algorithm^39^ was employed to select the most relevant features while minimizing redundancy among them. This method ensures that the selected features are highly informative and have a low correlation with each other. These techniques were applied to enhance the accuracy and computational efficiency of subsequent machine learning algorithms.

Finally, patches were labelled as positive or negative based on the percentage of annotation within the patch. For this classification task, four different machine learning algorithms were trained: K-Nearest Neighbors (KNN), Support Vector Machine (SVM), Random Forest (RF), and Artificial Neural Network (ANN).

### Multi-class segmentation using deep learning

**Figure 4:**
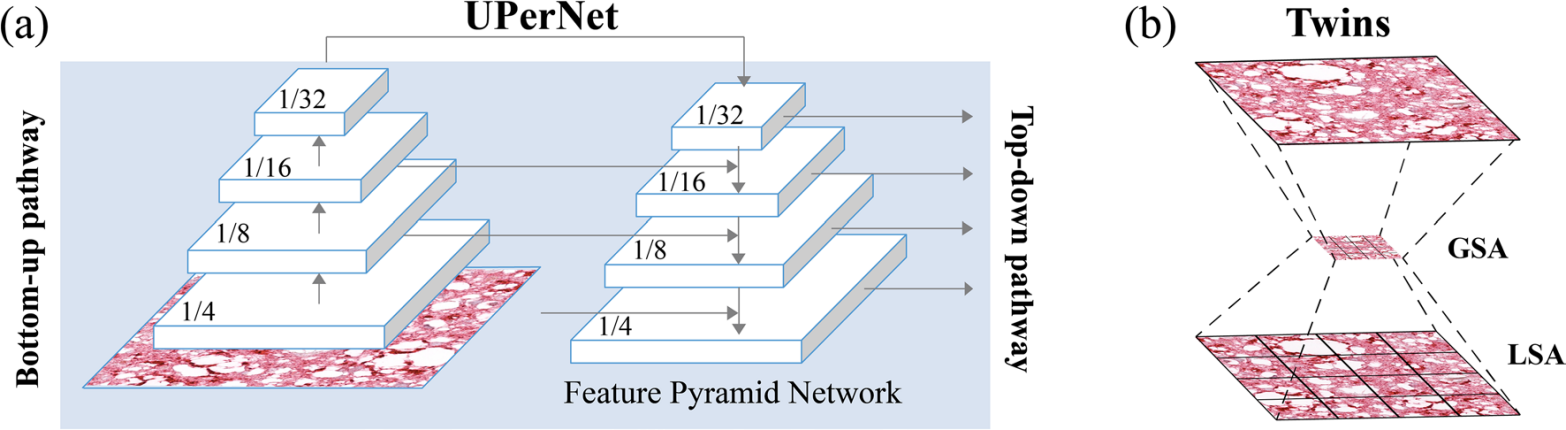
Segmentation network used in this work. (**a**) UPerNet is based on Feature Pyramid Network that extract image features at different scales. (**b**) Twins interleaves locally grouped attention (LSA) with global sub-sampled attention (GSA) during feature extraction.

To identify positive and negative patterns, we adopted a recent architecture based on vision transformers, an emerging category of deep learning networks^40,41^. Specifically, we integrated a vision transformer into the feature extraction phase of a UPerNet^42^. The UPerNet architecture is a widely used network that combines encoding and decoding layers with skip connections and multi-scale fusion to generate accurate and detailed segmentation maps. This network is based on the Feature Pyramid Network (FPN)^43^, which performs predictions at different scales instead of only on the finest level. The UPerNet employs lateral connections to fuse feature maps generated by the bottom-up pathway, the feed-forward computation of the backbone, with those of the top-down pathway, obtained by upsampling feature maps from the top to the base of the pyramid (Fig. 4a). This results in a set of proportionally sized feature maps at multiple levels. Skip connections are used to preserve fine-grained details in the segmentation map, while the multi-scale fusion module fuses feature maps from different decoder and corresponding encoder layers using a weighted sum.

To enhance the multi-scale feature extraction of the UPerNet, we used the Twins Transformer^44^ as the backbone. The Twins architecture extracts local features from image patches and selectively focuses on important regions within each patch through spatially variant attention mechanisms. The Twins employs locally grouped self-attention modules (LSA) and global sub-sampled attention modules (GSA) (Fig. 4b). LSA operates on a local patch, divided into a grid of tokens, and performs self-attention across the tokens within that patch. This allows the model to analyze the local features of the patch while preserving its spatial structure^44^. GSA, on the other hand, operates on a global representation of the image by subsampling the feature map. Both LSA and GSA allow the Vision Transformer to capture complex and multi-scale patterns within an image. By combining these two types of attention mechanisms, the model can selectively focus on important regions of the image at multiple scales, leading to more accurate and robust feature extraction.

During the training phase, we employed a batch size of 4 and an initial learning rate of 10^−4^, with a total of 100 epochs. To prevent overfitting, real-time random flipping was applied. We utilized Adam's optimization algorithm with a weight decay of 0.005, and both the decode and auxiliary head of the model employed a weighted cross-entropy loss (Eq. 2). The weights *W*_*c*_ of the cross-entropy loss were assigned inversely proportional to the number of pixels belonging to class *c*, ensuring that the least represented class (i.e., positive patterns) would have a higher contribution during the weight update compared to the more represented class (i.e., negative patterns). The loss is defined as:

2 $$ L_{{W_{CE} }} = - \frac{1}{N}\mathop \sum \limits_{c = 1}^{C} W_{c} \cdot \mathop \sum \limits_{i = 1}^{N} g_{ic} \cdot \log \left( {p_{ic} } \right) $$

The terms *p*_*ic*_ and *g*_*ic*_ represent the predicted segmentation probability and the ground truth label of class *c* at pixel *i*, respectively. *N* and *C* are the numbers of pixels and classes in the training dataset.

### Quantification of protein expression

The method for detecting prion protein expression can be applied to the entire WSI in order to extract quantitative features that are relevant to pathologists. Once the AI generates a binary mask, two options become available: 3D quantification in the brain and 2D quantification on the slide.

**Figure 5:**
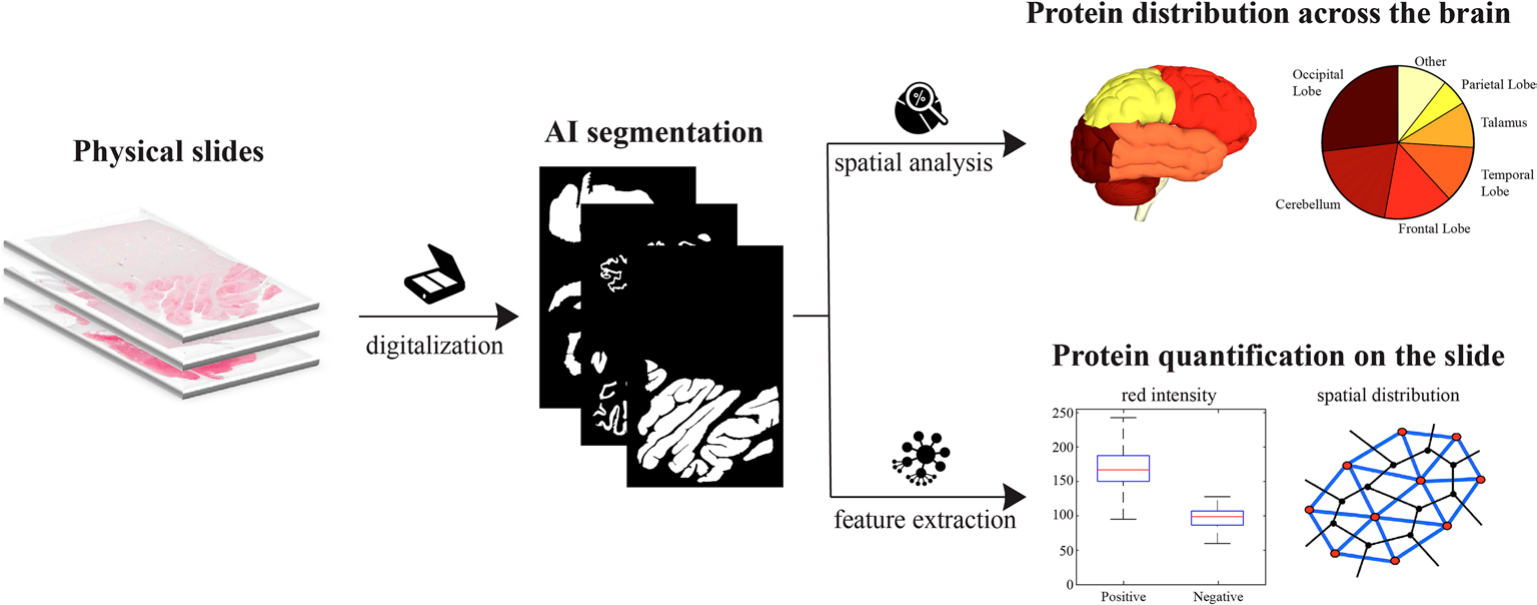
The proposed method employs a binary mask generated by AI to quantify protein expression across the WSIs. This analysis includes 3D assessment across the entire brain and 2D evaluation on individual slides.

In the first scenario, the software can determine the presence of the protein on the slide by calculating the ratio of positive tissue to the total histological tissue, based on known brain regions (such as the cerebellum, occipital cortex, etc.). When extensive sampling covers multiple brain regions, our algorithm can reconstruct the protein levels for each specific area, providing a comprehensive spatial distribution analysis across the entire brain (first branch in Fig. 5).

Alternatively, if the objective is to quantify protein expression on the current slide, the software enables the extraction of various quantitative features. These features include texture operators (as detailed in section "Feature extraction and classification using machine learning") and spatial distribution characteristics such as Voronoi diagrams (reference). This allows for quantifying both the intensity and spatial distribution of the prion protein on the individual slide, delivering valuable quantitative data to speed up the diagnostic process for pathologists (second branch of Fig. 5).

### Performance metrics

To evaluate the system's ability to recognize histological patterns in individual patches, we calculated the accuracy for the machine learning classifiers and the dice coefficient (DSC) for the segmentation network. For the entire WSI classification, we compared the automatic label (e.g., positive, or negative slide) with that provided by the expert operator.

## Results

### Machine learning performance

We conducted tests on the four traditional classifiers (KNN, SVM, RF, ANN) by adjusting their input conditions and hyperparameters. Features were extracted from grayscale images, DeltaE transforms, or a combination of both (Grayscale + DeltaE). Additionally, we changed the normalization type (min–max scaling or z-score) and the number of features (using PCA or mRMR) to determine the optimal set of descriptors. We adjusted the hyperparameters of the classifiers by varying the k-parameter and distance metric of KNN, the penalty parameter C and kernel trick for SVM, the number of trees and splitting criterion for RF, and the hyperparameters of ANN, such as batch size or number of hidden neurons. To analyze the results, we grouped all trials based on the input image used and created Table 2 to display the best accuracy achieved by each classifier.

**Table 2:** Patch-level performance of the machine learning approaches.

Classifier	Image	ACC_TRAIN_	ACC_VAL_ (%)
KNN	Grayscale	95.0	90.5
	DeltaE	96.5	92.8
	Grayscale + DeltaE	96.8	93.1
SVM	Grayscale	99.3	95.8
	DeltaE	98.5	96.8
	Grayscale + DeltaE	99.2	97.4
RF	Grayscale	100	93.3
	DeltaE	100	94.7
	Grayscale + DeltaE	100	95.1
ANN	Grayscale	98.4	96.0
	DeltaE	99.7	97.4
	Grayscale + DeltaE	99.5	**97.6**

Each of the four classifiers (KNN, SVM, RF, and ANN) are trained and tested on three different types of images: Grayscale, DeltaE and the combination of both (Grayscale + DeltaE). Best results on validation set are highlighted in bold.

**Figure 6:**
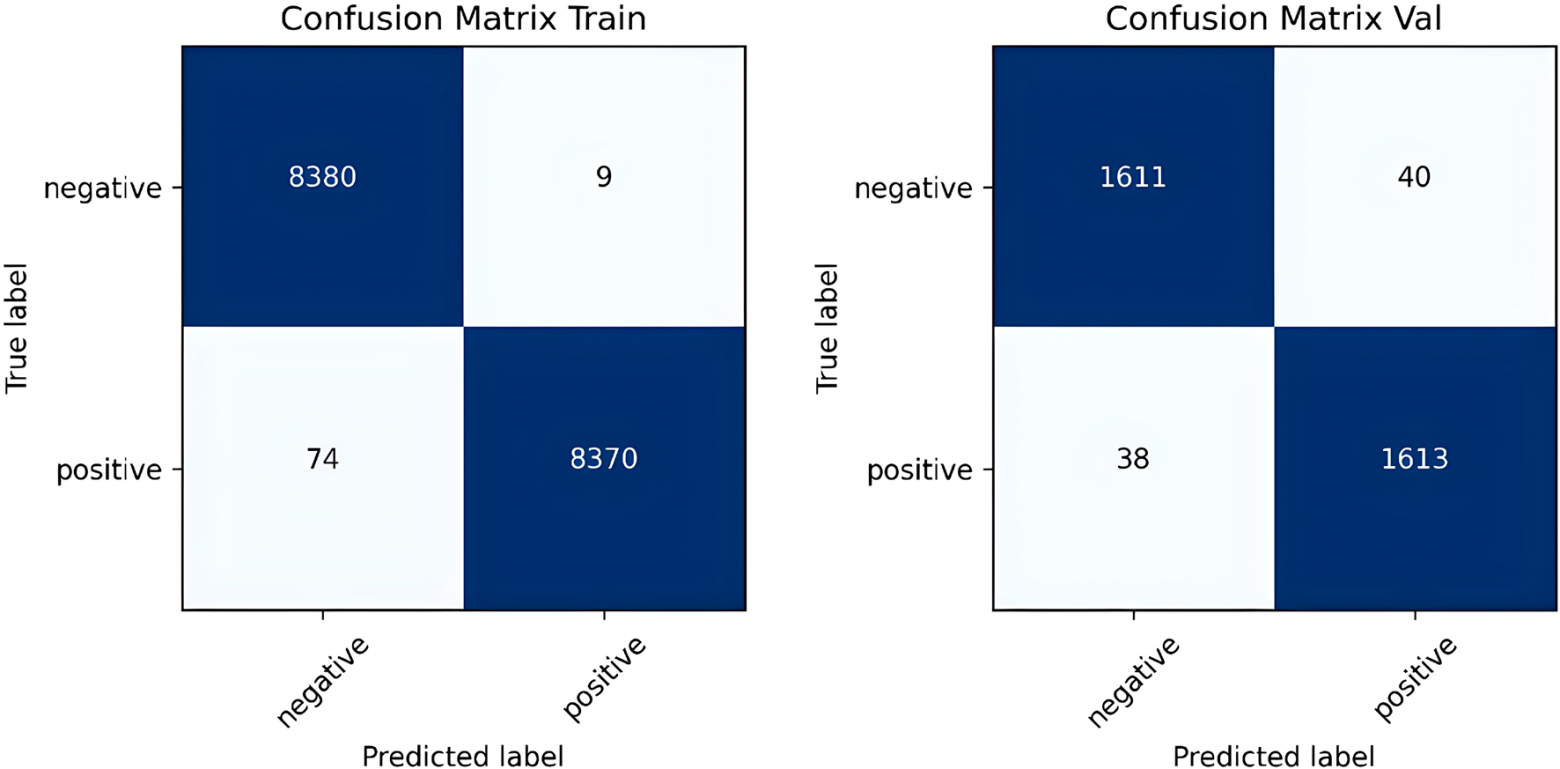
Confusion matrixes of the best performing machine learning classifier (ANN) on train and validation set.

All the classifiers achieved an accuracy of over 90% for both the training and validation sets across each of the three types of input features. The DeltaE transform was found to be more informative than the traditional grayscale image. However, the combination of features extracted from both images produced the best results. The best-performing classifier on the validation set was the ANN, which achieved an average accuracy of 97.6%. The ANN consisted of two hidden layers, each containing 800 neurons. It was trained for 100 epochs with a batch size of 256 and a learning rate of 0.0001. Z-score normalization was applied to the features extracted from the grayscale image and DeltaE. The confusion matrices shown in Fig. 6 demonstrate that the performance on the two subsets were well-balanced. In fact, the validation set misclassifications included 78 out of 3302 patches, comprising 40 false positives and 38 false negatives.

### Deep learning performance

**Figure 7:**
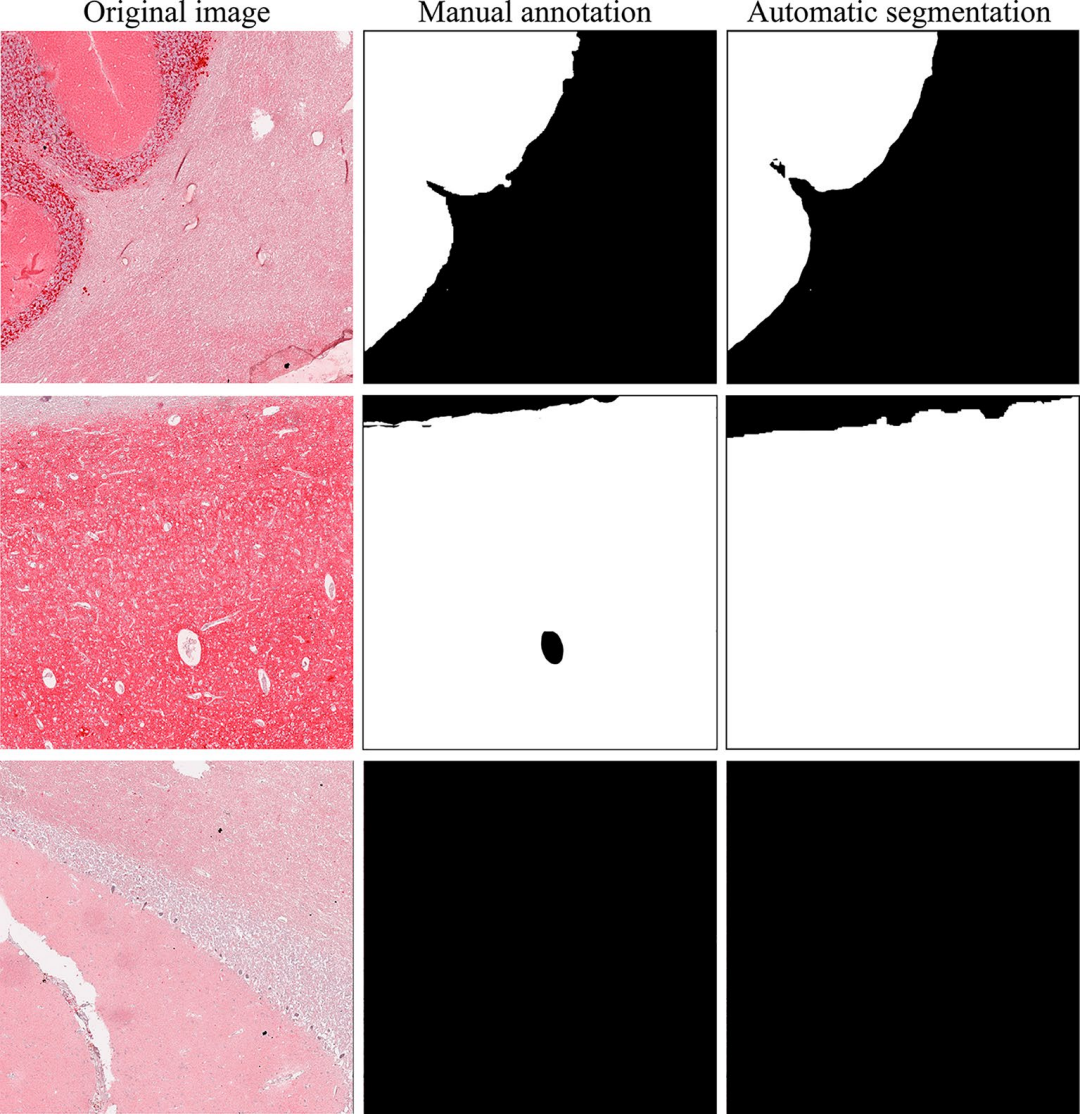
Performance of the deep learning-based method in the segmentation of PrPSc aggregates in three different tiles.

The UPerNet-Twins (Fig. 4) adopted in this study focused on segmenting histopathological patterns rather than classifying input patches. The idea was to identify areas of the tissue sample that exhibited distinct patterns of staining, which could be indicative of the presence of PrPSc aggregates. As a result, two different segmentation metrics were utilized: Dice similarity coefficient (DSC) and accuracy. These metrics were used to evaluate the system's ability to identify PrPSc aggregates. The UPerNet-Twins network performed consistently well on both the training and validation sets, achieving a DSC of 73.7% and 70.2%, respectively, and an accuracy of 93.6% and 93.7%. A visual comparison between manual annotation and the proposed network segmentation is shown in Fig. 7.

### Comparison of the two approaches for WSI labeling

**Figure 8:**
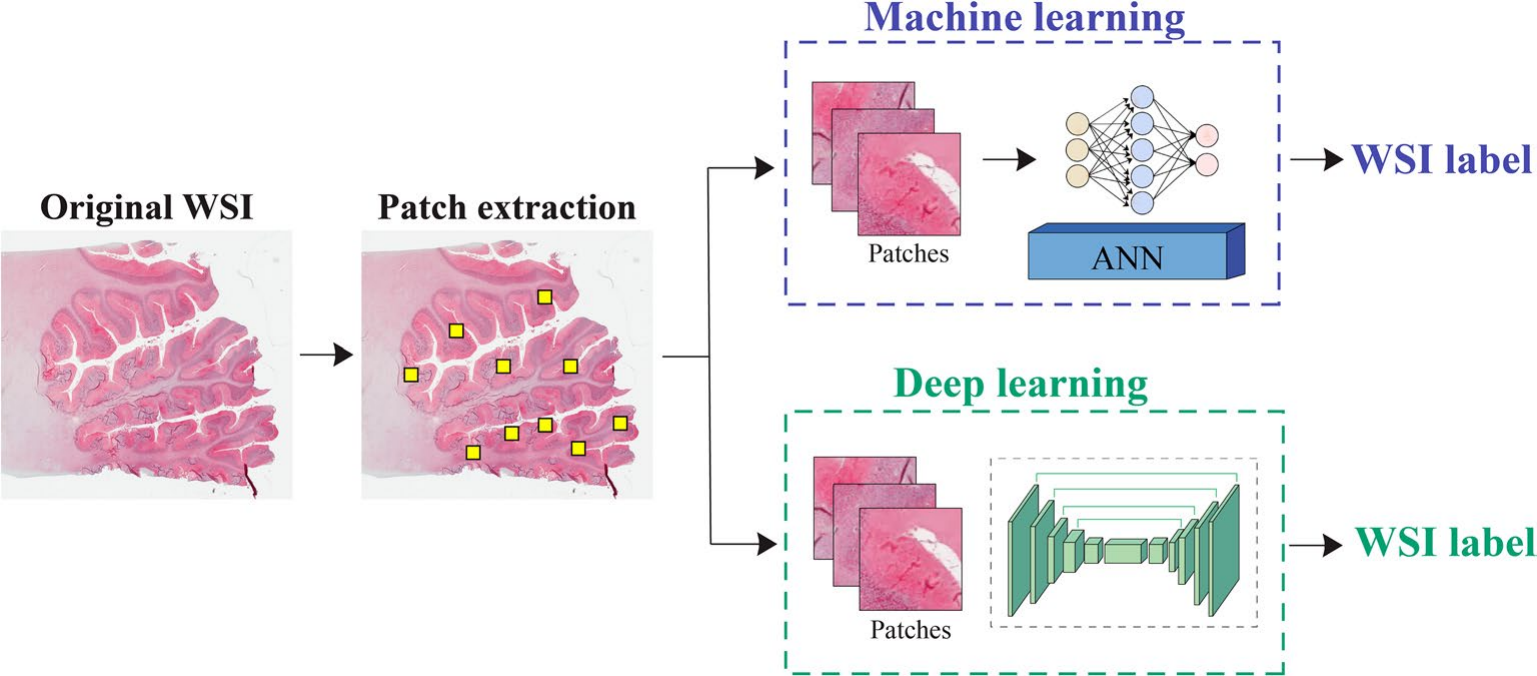
Comparison in WSI labelling for machine-learning and deep learning-based methods. The process involves extracting relevant patches using a threshold on the red channel of the original WSI (depicted as yellow squares). The Artificial Neural Network (ANN) performs patch classification, while UPerNet-Twins employs a segmentation paradigm. Finally, a threshold on positive pixels is applied to obtain the final label of the slide.

To evaluate the performance of the machine learning and deep learning-based methods, we utilized WSIs, which offer a comprehensive view of the entire tissue sample. Information on WSI is generally stored in high-resolution patches, which makes it time-consuming to analyze an entire slide. To address this challenge, we developed a smart patch extraction approach that identifies the most diagnostically relevant portion of tissue. Our method involves applying a threshold in the RGB channel and extracting patches with a red intensity greater than the 90th percentile of the entire slide. This approach significantly speeds up WSI inference by up to 700% while maintaining a high level of accuracy. Figure 8 provides a visual representation of the effectiveness of this method.

For the machine learning-based approach, patch classification results were extrapolated to the entire WSI by calculating the percentage of pixels labeled as pathological among all pixels containing a portion of the tissue. If this percentage exceeded 10%, the slide was labeled as positive for pathology. This threshold was chosen based on empirical data analysis and was found to be an appropriate cutoff for accurate classification.

Similarly, for the deep learning-based approach, segmentation network was applied to the WSI using a sliding window approach. The same threshold used in the machine learning-based approach was applied to the resulting heatmap to label the slide as positive or negative for pathology. Figure 8 illustrates the WSI labeling procedure of the proposed method. In addition to evaluating the accuracy of the machine learning and deep learning-based methods, we also assessed the number of patients correctly classified using each approach (Table 4). As can be seen, the best machine learning-based approach (ANN) misclassified 2 patients in the training set, 1 in the validation set, and 2 in the testing set. The deep learning-based approach (UPerNet-Twins), on the other hand, misclassified only 1 patient in the training and validation sets and correctly classified all patients in the testing set.

## Discussion

In this research paper, we have introduced an innovative and automated pipeline that employs AI-based algorithms to analyze WSIs and classify them as either positive or negative for prion disease. To the best of our knowledge, this is the first research work that employs machine learning and deep learning techniques for analyzing Whole Slide Images (WSIs) related to prion diseases. We also present a new type of image (DeltaE transform) that allows to extract more informative features and improves the classification performance of traditional machine learning techniques. In addition, we propose a multi-scale architecture based on a vision transformer^44^ capable of segmenting the histological pattern associated with prion diseases.

The metrics computed during the construction process for both ML and DL approaches showed that both methods were suitable for this task (Tables 2 and 3). However, in a blind test set (Table 4), the DL method demonstrated superior performance compared to the best-performing ML method (ANN). These results suggest that the DL approach has significant advantages over the traditional ML method in the context of prion disease diagnosis. By utilizing more complex algorithms and neural network architectures, DL can more accurately identify patterns and anomalies in WSIs, which is particularly crucial in the detection of prion diseases. Hence, the Twins-UPerNet proved to have better learned the characteristics of PrPSc aggregates with respect to the ANN.

**Table 3:** Patch-level performance of the deep learning approach.

Method	Subset	DSC (%)	ACC (%)
UPerNet-Twins	Train	73.7 ± 9.1	93.6 ± 8.5
	Val	70.2 ± 7.5	93.7 ± 6.8

The performance of the network was assessed in terms of the Dice coefficient (DSC) and accuracy (ACC) for both the training and validation sets.

**Table 4:** Performance comparison between the best machine-learning approach (ANN) and the deep learning-based method (UPerNet-Twins) for WSI labelling in the training, validation, and test sets.

Method	Subset	WSI correctly classified
ANN	Train	47/49
	Val	8/9
	Test	4/6
UPerNet-Twins	Train	48/49
	Val	8/9
	Test	6/6

Training the deep neural network took longer and required more resources, but once the system was trained, its application time was considerably lower than that of the ANN (31 s vs. 95 s). In fact, the use of texture analysis in feature extraction for the ML approach had a significant impact on computational time, making it a time-consuming process. One of the benefits of using deep learning was the higher accuracy in delineating histopathological patterns. This was achieved by a segmenter, which allowed for pixel-level precision. In summary, the proposed deep neural network offered several advantages over traditional classifiers, including:

- Extraction of more informative features
- Reduced computation time
- Improved accuracy in outlining the shape of PrPSc aggregates.

Applying this approach to extensive tissue samples could be highly beneficial in clinical and pathological practice. It could provide valuable insights into the distribution and spatial localization of PrPSc throughout the entire brain, leading to improved classification of prion disease cases (Fig. 5). Furthermore, this technique has the potential to be extended to evaluate multiple proteinopathies in other neurodegenerative diseases, including Alzheimer's and Parkinson's diseases.

The main limitation of this study was the restricted variance in the cases examined, primarily due to the rarity of prion diseases. Consequently, we had to rely on tissue slides from a single specialized medical center—namely, the Maria Vittoria Hospital of Turin—and annotations from a single pathologist. While this approach ensured consistency in the acquisition and processing of WSIs, it also introduced a possible source of bias in the data. To enhance the generalizability of our findings, future research should aim to incorporate WSIs from multiple medical centers, thereby increasing the variability in staining across slides, and involve a consensus among multiple pathologists for data annotation. In addition, incorporating other phenotypes of prion diseases would provide a more comprehensive understanding of the diagnostic capabilities of the proposed DL algorithm.

In our study, we also acknowledge the limitations associated with staining for synaptic deposits. The variability in immunostaining for synaptic types across different cases can make it challenging to determine and categorize these plaques accurately (see Supplementary Materials). We recognize the need to improve our approach to account for these staining variabilities in future work. Some potential strategies include incorporating uncertainty estimates in the model outputs to identify low-confidence synaptic deposit detections for expert pathologist review and focusing on developing a semi-quantitative grading schema or a machine learning approach to better characterize the level of positivity. These advancements would enhance the accuracy and reliability of our AI-powered pathology framework for prion disease diagnosis.

The present study only included slides from two anatomical regions—the occipital lobe of the cerebral cortex and the cerebellum. Thus, future studies should include tissue samples from a wider range of regions to increase the generalizability of the findings. A larger dataset would also enable the development of a model capable of distinguishing between different types of PrPSc aggregates. This would provide a second quantitative opinion about the presence of prion disease in the patient and yield additional diagnostic information to help identify the specific phenotype of the disease.

## Conclusion

This paper presents a novel approach for the automated classification of WSIs as positive or negative for prion disease. Our proposed method utilizes advanced AI techniques to accurately detect patterns and anomalies in the WSIs, facilitating the diagnosis of prion disease with improved accuracy and speed. In addition, our tool can be utilized for quantitative analysis of extended brain samples and applied to other neurodegenerative diseases.

### Supplementary Information

Supplementary Information.

## Data Availability

The digitized slides, manual annotations, and code used in this study are available from the corresponding author upon reasonable request.

## References

[1] Das AS, Zou W-Q (2016). Prions: Beyond a single protein. Clin. Microbiol. Rev..

[2] Morales R (2016). Strain-dependent profile of misfolded prion protein aggregates. Sci. Rep..

[3] Rossi M, Baiardi S, Parchi P (2019). Understanding prion strains: Evidence from studies of the disease forms affecting humans. Viruses.

[4] Lee J (2015). Laboratory diagnosis and surveillance of Creutzfeldt–Jakob disease. J. Med. Virol..

[5] Cali I (2006). Classification of sporadic Creutzfeldt–Jakob disease revisited. Brain.

[6] Budka H, Jellinger Kl (1995). Neuro-pathological diagnostic criteria for CreutzfeldtJakob disease (CJD) and other human spongiform encephalopathies (prion diseases). Brain Pathol..

[7] Nishio M, Nishio M, Jimbo N, Nakane K (2021). Homology-based image processing for automatic classification of histopathological images of lung tissue. Cancers.

[8] Rexhepaj E (2013). A texture based pattern recognition approach to distinguish melanoma from non-melanoma cells in histopathological tissue microarray sections. PLoS ONE.

[9] Alinsaif S, Lang J (2020). Texture features in the Shearlet domain for histopathological image classification. BMC Med. Inform. Decis .Mak..

[10] Trivizakis E (2021). A neural pathomics framework for classifying colorectal cancer histopathology images based on wavelet multi-scale texture analysis. Sci. Rep..

[11] Haghighat M (2022). Automated quality assessment of large digitised histology cohorts by artificial intelligence. Sci. Rep..

[12] Takamatsu M (2022). Prediction of lymph node metastasis in early colorectal cancer based on histologic images by artificial intelligence. Sci. Rep..

[13] Salvi M (2021). Histopathological classification of canine cutaneous round cell tumors using deep learning: A multi-center study. Front. Vet. Sci..

[14] Salvi M (2021). Automated assessment of glomerulosclerosis and tubular atrophy using deep learning. Comput. Med. Imaging Graph..

[15] Laury AR, Blom S, Ropponen T, Virtanen A, Carpén OM (2021). Artificial intelligence-based image analysis can predict outcome in high-grade serous carcinoma via histology alone. Sci. Rep..

[16] Salvi M, Cerrato V, Buffo A, Molinari F (2019). Automated segmentation of brain cells for clonal analyses in fluorescence microscopy images. J. Neurosci. Methods.

[17] Wang C-W (2021). Artificial intelligence-assisted fast screening cervical high grade squamous intraepithelial lesion and squamous cell carcinoma diagnosis and treatment planning. Sci. Rep..

[18] Singh R, Wu W, Wang G, Kalra MK (2020). Artificial intelligence in image reconstruction: The change is here. Phys. Med..

[19] Salvi M (2020). Karpinski score under digital investigation: A fully automated segmentation algorithm to identify vascular and stromal injury of Donors’ kidneys. Electronics.

[20] Zhao, Y. & Li, X. Research on the application of artificial intelligence in medical imaging diagnosis. in *2022 Global Conference on Robotics, Artificial Intelligence and Information Technology (GCRAIT)* 237–241 (IEEE, 2022).

[21] Borghammer P (2018). How does Parkinson’s disease begin? Perspectives on neuroanatomical pathways, prions, and histology. Move. Disord..

[22] Kothari S, Phan JH, Stokes TH, Wang MD (2013). Pathology imaging informatics for quantitative analysis of whole-slide images. J. Am. Med. Inform. Assoc..

[23] Van der Velden BHM, Kuijf HJ, Gilhuijs KGA, Viergever MA (2022). Explainable artificial intelligence (XAI) in deep learning-based medical image analysis. Med. Image Anal..

[24] Saleem TJ (2022). Deep learning-based diagnosis of Alzheimer’s disease. J. Pers. Med..

[25] Chang C-H, Lin C-H, Lane H-Y (2021). Machine learning and novel biomarkers for the diagnosis of Alzheimer’s disease. Int. J. Mol. Sci..

[26] Pagano G, Niccolini F, Politis M (2016). Imaging in Parkinson’s disease. Clin. Med..

[27] Jin L (2021). Artificial intelligence neuropathologist for glioma classification using deep learning on hematoxylin and eosin stained slide images and molecular markers. Neuro Oncol..

[28] Signaevsky M (2019). Artificial intelligence in neuropathology: Deep learning-based assessment of tauopathy. Lab. Invest..

[29] Bhakta, A. & Byrne, C. Creutzfeldt–Jakob disease prediction using machine learning techniques. in *2021 IEEE 9th International Conference on Healthcare Informatics (ICHI)* 535–542 (IEEE, 2021).

[30] Bizzi A (2020). Evaluation of a new criterion for detecting prion disease with diffusion magnetic resonance imaging. JAMA Neurol..

[31] Kovács GG (2002). Immunohistochemistry for the prion protein: comparison of different monoclonal antibodies in human prion disease subtypes. Brain Pathol..

[32] Gillies RJ, Kinahan PE, Hricak H (2016). Radiomics: Images are more than pictures, they are data. Radiology.

[33] Corrias G, Micheletti G, Barberini L, Suri JS, Saba L (2022). Texture analysis imaging “what a clinical radiologist needs to know”. Eur. J. Radiol..

[34] Davnall F (2012). Assessment of tumor heterogeneity: An emerging imaging tool for clinical practice?. Insights Imaging.

[35] Tomita F, Tsuji S, Tomita F, Tsuji S (1990). Statistical texture analysis. Comput. Anal. Vis. Text..

[36] Galloway MM (1975). Texture analysis using gray level run lengths. Comput. Graph. Image Process..

[37] Pietikäinen M (2010). Local binary patterns. Scholarpedia.

[38] Jolliffe, I. T. *Principal Component Analysis*, 2nd edn (Springer, 2002).

[39] De Jay N (2013). mRMRe: An R package for parallelized mRMR ensemble feature selection. Bioinformatics.

[40] Vaswani A (2017). Attention is all you need. Adv. Neural Inf. Process. Syst..

[41] Lin T, Wang Y, Liu X, Qiu X (2022). A survey of transformers. AI Open.

[42] Xiao, T., Liu, Y., Zhou, B., Jiang, Y. & Sun, J. Unified perceptual parsing for scene understanding. in *Proceedings of the European conference on computer vision (ECCV)* 418–434 (2018).

[43] Lin, T.-Y. *et al.* Feature pyramid networks for object detection. in *Proceedings of the IEEE Conference on Computer Vision and Pattern Recognition* 2117–2125 (2017).

[44] Chu X (2021). Twins: Revisiting the design of spatial attention in vision transformers. Adv. Neural Inf. Process. Syst..

