# Supplementary material for: Quantitative analysis of prion disease using an AI-powered digital pathology framework
Source: Sci Rep. 2023 Oct 18;13:17759. doi: 10.1038/s41598-023-44782-4 (PMC10584956; doi:10.1038/s41598-023-44782-4)
Supplement: Supplementary file 1 — Supplementary Information. [file 41598_2023_44782_MOESM1_ESM.docx]

Supplementary materials of the paper: **Quantitative Analysis of Prion Disease using an AI-Powered Digital Pathology Framework**, *Massimo Salvi, Filippo Molinari, Mario Ciccarelli, Roberto Testi, Stefano Taraglio, Daniele Imperiale*, Scientific Reports, 2023

**Tiles extraction and smart patch extraction**

Macro-tiles with dimensions 3072x3072x3 at 20x magnification are extracted from WSIs to construct the datasets, as they are easier to manage compared to the entire WSI. To accomplish this, a tile iterator is implemented using the Python library large_image ^1^. The iterator takes as input the starting pixel, the dimensions of the tiles to extract, and the overlap between consecutive tiles. Sliding over the entire image, the iterator ensured that there was no overlap between tiles (Figure S1).

Each extracted tile is then analyzed to determine if it contained a certain percentage of tissue labeled as either positive or negative, surpassing a pre-set threshold. The threshold is intentionally set to a low value (10%) to minimize the risk of discarding potentially useful tissue during the tile extraction process.


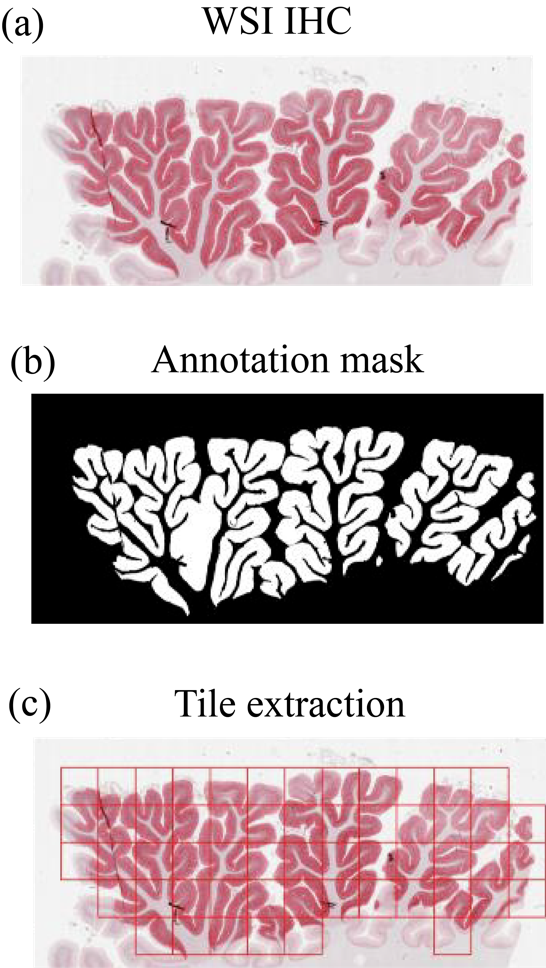


**Figure S1.** Steps followed during tile extraction. (a) original WSI with IHC staining, (b) corresponding binary segmentation mask, (c) location of the extracted tiles (red rectangles).

To construct the dataset of images used as input for the machine learning (ML) and deep learning (DL) algorithms, a sliding window is employed to extract smaller patches with dimensions of 512x512x3. The number of patches extracted from each WSI is limited to a maximum of 500, as there are more positive cases (n=48) compared to negative controls (n=16). This selection criterion helps balance the dataset by ensuring an equal representation of positive and negative patches for prion diseases. Figure S2 illustrates the percentage of 'positive' versus 'negative' patches after this smart patch extraction, demonstrating the successful balancing of the dataset despite the fewer control cases.


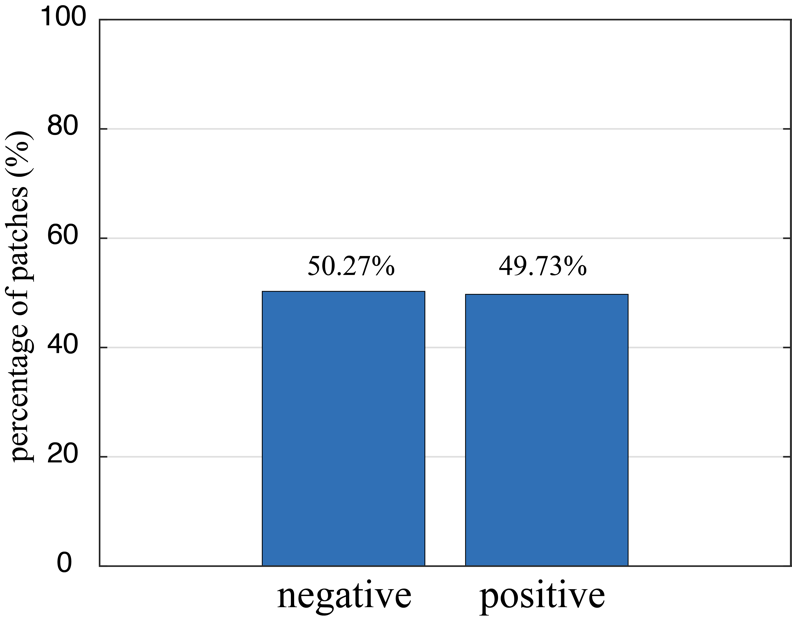


**Figure S2.** Percentage of patch labeled as ‘positive’ and ‘negative’ after patch selection.

Slightly different strategies are employed for the ML and DL approaches. In the case of ML, a patch is considered eligible for extraction only if it contains at least 95% annotated tissue. This criterion is implemented since the ML approach focuses on classification, where completely positive or negative patches were desired. On the other hand, since the DL approach involves a segmentation task, all extracted patches are utilized. Table S1 provides the number of patches used in the construction set for both the ML and DL approaches, indicating a substantial number of patches for both ML (total: 20,135) and DL (total: 32,886).

**Table S1**

Number of extracted patches for the ML and DL approach.

|  | **Number of patches extracted** | |
| --- | --- | --- |
| **Method** | **Train** | **Val** |
| ML | 16833 | 3302 |
| DL | 28403 | 4483 |

**Suboptimal and faulty cases**

The network demonstrates excellent recognition performance on clear, intense patterns, particularly for synaptic patterns with varying intensities as shown in Figure 7 and Figure S3a. However, the network struggles with weak patterns, as evidenced in Figure S3b, where the network correctly identifies overall tissue positivity but does not accurately segment the full positive area.


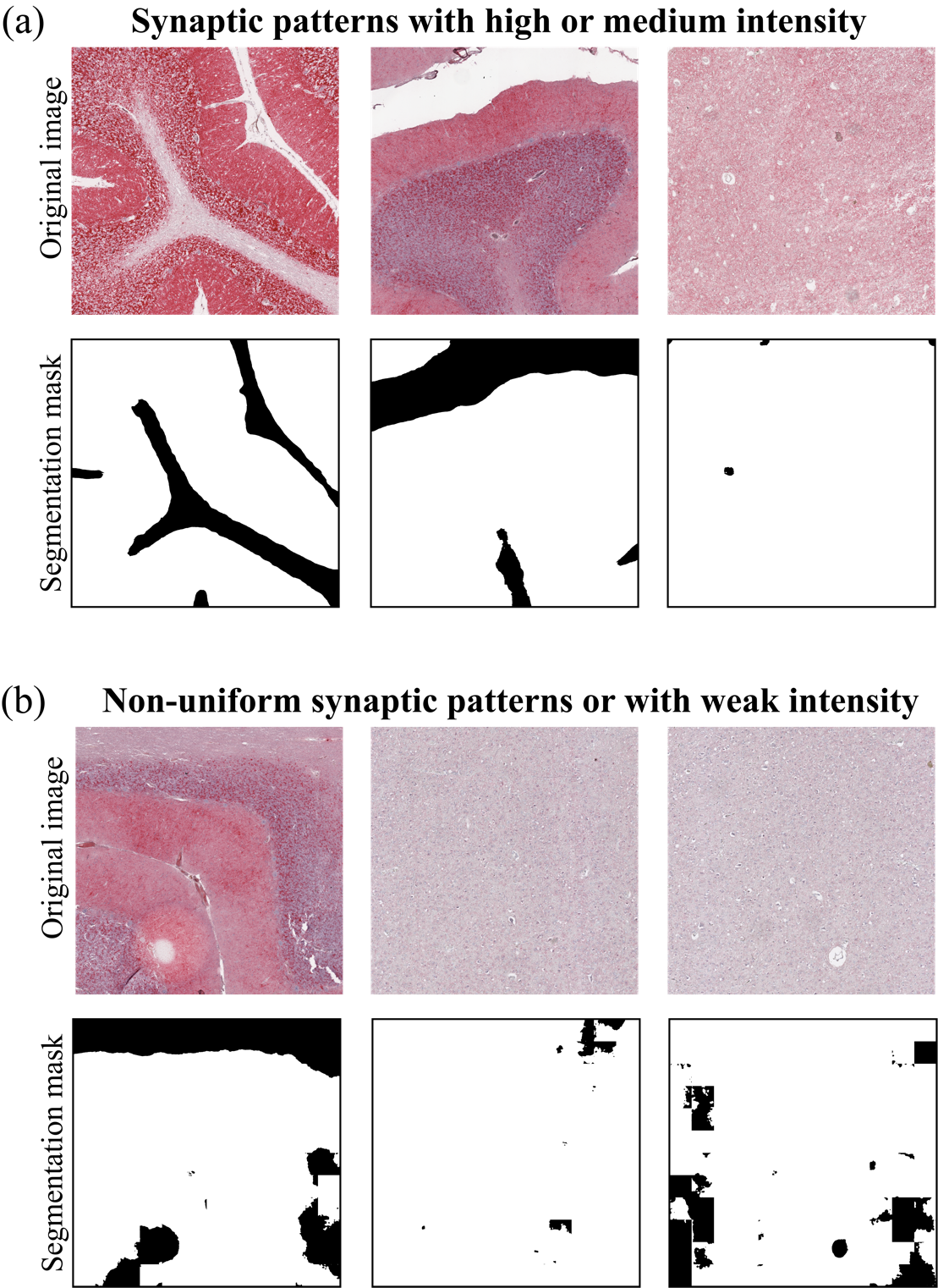


**Figure S3**  **.** Example of tiles with different synaptic patterns. (a) Images with high or medium intensity. (b) Images with non-uniform synaptic patterns or with weak intensity.

In other cases, the network recognizes the presence of positivity, but the probability map does not reach the threshold required to identify the entire positive tissue area, as shown in Figure S4. Specifically, the network does not identify weak patterns as uniform. Importantly though, these challenging areas did not span the entirety of the WSIs in our experiments. Using the proposed patch aggregation approach for WSI labeling (Section 3.3), these localized errors did not significantly impact the final WSI classification.

It is notable that the network identifies difficult patterns that are challenging even for a human operator to discern, either due to weak staining or subtle visual features. Addressing these hard examples could be a target for future improvement.


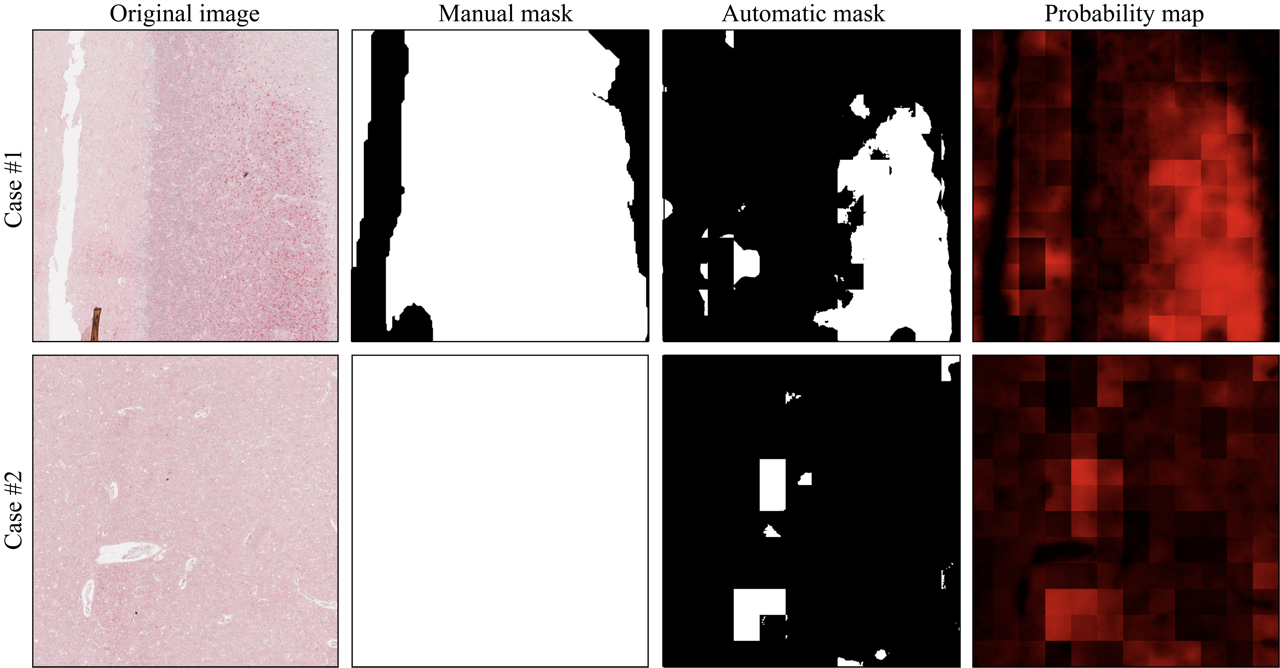


**Figure S4**  **.** Examples of images with weak and non-uniform synaptic patterns. The network identifies tissue positivity through the probability map but lacks sufficient confidence to include it within the automatic mask.

References:

[1] *Kitware, «large-image - PyPI,» [Online]. Available: https://pypi.org/project/large-image/*
